# In Response

**DOI:** 10.4269/ajtmh.2011.10-0694b

**Published:** 2011-03-04

**Authors:** John A. Crump, Jeremy Sugarman

**Affiliations:** Division of Infectious Diseases and International Health; Duke University Medical Center; Durham, NC 27710; E-mail: crump017@mc.duke.edu; Berman Institute of Bioethics and Department of Medicine; Johns Hopkins University; Baltimore, MD 21205; E-mail: jsugarm1@jhmi.edu

Dear Sir:

The concerns raised by Garcia and others^1^ about reciprocity in global health training programs are critically important. Lack of consistent efforts to establish mutual and reciprocal benefits in global health training programs is a major ethical concern^2^ and was one of the observations that led to the formation of the Working Group on Ethics Guidelines for Global Health Training (WEIGHT). As indicated by Garcia and others, although the ethics and best practice guidelines for training experiences in global health^3^ were designed to apply to reciprocal training programs, the guideline's development was catalyzed predominantly by concerns observed when trainees from high-income settings seek training experiences in low-income settings. With some exemplary exceptions, global health trainees have predominantly flowed from high-income to low-income settings,^4^ thereby limiting the range of actual experiences available to help inform the development of the WEIGHT guidelines.

The WEIGHT guidelines summarize complex issues, each of which could be further elaborated. For example, although it is likely that most short-term trainees from high-income countries seeking short-term training experiences in low-income settings provide little benefit to the host, it is possible that such experiences could be nested in long-term collaborations that do. Such long-term collaborations may go beyond the development of reciprocal training opportunities championed by Garcia and others to also include activities that benefit the host in a wider range of ways, such as infrastructure development, research collaboration, or long-term service programs.

As should be clear, although there are strong conceptual arguments regarding the goals of global health programs, there is a lack of systematically collected data on the benefits and harms of global health training programs to hosts. Furthermore, there is a paucity of guidance available for those planning or engaged in global health training programs. However, the circumstance of growing enthusiasm about global health training experiences on a background of a range of ethical concerns compelled us to start somewhere. As mentioned in the initial publication of the guidelines, we welcome discussion, deliberation, dissemination, and descriptions of how and when the guidelines work and when they fail to do so. We fully envision revisions and improvements on the guidelines.
